# Diagnostic value of nucleic acid amplification tests on bronchoalveolar lavage fluid for smear-negative pulmonary tuberculosis: a meta-analysis

**DOI:** 10.1042/BSR20140186

**Published:** 2015-07-29

**Authors:** Panwen Tian, Yongchun Shen, Ye Wang, Chun Wan, Mei Feng, Jing Zhu, Ting Yang, Lei Chen, Fuqiang Wen

**Affiliations:** * Department of Respiratory and Critical Care Medicine, West China Hospital of Sichuan University and Division of Pulmonary Diseases, State Key Laboratory of Biotherapy of China, Chengdu 610041, China

**Keywords:** bronchoalveolar lavage, diagnosis, meta-analysis, nucleic acid amplification test, smear-negative pulmonary tuberculosis

## Abstract

The diagnosis of smear-negative pulmonary tuberculosis (SNPT) remains a clinical challenge. Many studies suggest that nucleic acid amplification tests (NAATs) on bronchoalveolar lavage fluid (BALF) plays a role in diagnosing SNPT, but with considerable varying results. The current study aimed to summarize the overall diagnostic accuracy of NAATs assay on BALF for SNPT. A systematic literature search was performed and data were retrieved. Pooled sensitivity, specificity, positive likelihood ratio (PLR), negative likelihood ratio (NLR) and diagnostic odds ratio (DOR) were calculated. A summary receiver operating characteristic curve and area under the curve (AUC) were used to evaluate the overall diagnostic performance. All the statistical analysis was performed by using STATA 12.0 and Meta-DiSc 1.4 software. A total of nine studies with 1214 subjects were included this meta-analysis. The pooled sensitivity, specificity, PLR, NLR and DOR were 0.54 [95% CI (confidence interval): 0.48–0.59], 0.97 (95% CI: 0.95–0.98), 12.13 (95% CI: 8.23–17.88), 0.36 (95% CI: 0.23–0.56) and 44.71 (95% CI: 22.30–89.63) respectively. The AUC was 0.96. Estimated positive and negative post-probability values for a SNPT prevalence of 20% were 82% and 7% respectively. No publication bias was identified. Current available evidence indicated that NAATs on BALF may play a role in diagnosing SNPT, whereas the results should be interpreted in parallel with clinical information of patients and the results of traditional tests. Further studies should be performed to confirm our findings.

## Abstract (precis)

Based on current available evidence, NAAT on BALF plays a role in the diagnosis of smear-negative pulmonary tuberculosis, and further studies should be performed to confirm our findings..

## INTRODUCTION

Tuberculosis remains a major global health problem. According to World Health Organization's report, it is estimated that 8.6 million new tuberculosis cases occurred in 2012 worldwide and a substantial proportion were smear-negative pulmonary tuberculosis (SNPT) [1]. It was reported that there were 159121 cases of culture-confirmed pulmonary tuberculosis in the United States from 1993 to 2008, of which 58786 (37%) were sputum smear-negative [2]. Traditional diagnosis of pulmonary tuberculosis depends on sputum smear, which is rapid, specific and inexpensive but has low sensitivity ranging from 20% to 60%, when performed optimally [3], whereas sputum culture is time-consuming and chest imaging examination is with unsatisfactory result. SNPT is more difficult to diagnose and has been associated with poorer treatment outcomes and excessive mortality, particularly in high human immunodeficiency virus prevalent settings. The traditional practices of establishing pulmonary tuberculosis diagnosis are not sensitive and specific enough to confirm the diagnosis of SNPT, leading to missed diagnosis of pulmonary tuberculosis or overtreatment of people without pulmonary tuberculosis [4]. Thus, faster and more accurate diagnostic tests are required for better control of tuberculosis, especially for SNPT.

Nucleic acid amplification tests (NAATs) have been applied to serum, pleural effusions, bronchoalveolar lavage fluid (BALF), cerebrospinal fluids and bile to detect tuberculosis bacilli [5–8]. NAATs save the time required for the detection of the mycobacterium and probably enhance the identification of SNPT cases. The US Food and Drug Administration has approved the use of selective commercial NAATs for the detection of tuberculosis in both smear-positive and smear-negative specimens [9]. BALF is recommended for the diagnosis of pulmonary infectious diseases [10]. Use of NAATs on BALF may increase the sensitivity of detecting mycobacterial infection in patients with smear-negative sputum or those who are unable to produce enough sputum samples (sputum-scarce). In fact, several studies have been published on the potential diagnostic value of NAATs on BALF for SNPT or sputum-scarce tuberculosis, with varying results. Therefore, we performed a meta-analysis based on current available evidences to establish the overall accuracy of NAATs on BALF for SNPT or sputum-scarce tuberculosis.

## MATERIALS AND METHODS

This meta-analysis was carried out using the guidelines of the Preferred Reporting Items for Systematic Reviews, as well as the Meta-analysis Statement and methods recommended by the Cochrane Diagnostic Test Accuracy Working Group [11]. Institutional review board approval was not required for this retrospective meta-analysis.

### Search strategy and study selection

PubMed, Embase, Web of Science and the Cochrane database were searched to identify suitable studies regarding the diagnostic accuracy of NAATs for SNPT or sputum-scarce tuberculosis up to September, 2014. The main search terms were ‘polymerase chain reaction or PCR or nucleic acid amplification test’, ‘bronchoalveolar lavage or BALF’, ‘tuberculosis’, ‘sensitivity or specificity or accuracy’’. References of identified articles or review articles were also searched manually to identify potential studies.

A study was included if it met the following inclusion criteria: (1) It should be a diagnostic study that investigates the accuracy of NAATs on BALF for SNPT or sputum-scarce tuberculosis in humans; (2) both the sensitivity and specificity are provided or could be calculated; (3) each group contains more than 10 patients to avoid selection bias; (4) it should be published in English. Abstracts or meeting proceedings were excluded because of the limited data. Two reviewers independently judged study eligibility while screening the citations. In case of disagreement, the two reviewers arrived at a consensus.

### Data extraction and quality assessment

The final set of articles was assessed independently by two reviewers. Data retrieved from these articles included author, publication year, country of origin, tuberculosis diagnostic standard, patient number, test method and data for two-by-two tables. The methodological quality of each included study was assessed by using Quality Assessment for Studies of Diagnostic Accuracy (QUADAS) Checklist [12], an evidence-based approach for quality assessment in systematic reviews of diagnostic accuracy studies, which includes 14 items assessing risk of bias, sources of variation (applicability) and reporting quality; each item is rated ‘yes’, ‘no’, or ‘unclear’. The maximum value for each study is 14.

### Meta-analysis

The following indexes of test accuracy were computed for each study: sensitivity, specificity, positive likelihood ratio (PLR), negative likelihood ratio (NLR) and diagnostic odds ratio (DOR) with standard methods recommended for meta-analyses of diagnostic accuracy studies [13]. The inter-study heterogeneity was calculated by the chi-square-based Q test and the inconsistency index *I*^2^. When a significant Q test (*P*<0.05 or *I*^2^>50%) indicated heterogeneity among studies, the random-effect model was conducted; otherwise, the fixed-effect model was chosen. Meta-regression was performed to investigate the source of heterogeneity within the included studies. The summary receiver operating characteristic (SROC) curve was generated and the area under the curve (AUC) was calculated to summarize the overall diagnostic accuracy. Since publication bias is of concern for meta-analysis of diagnostic studies, we tested for the potential presence of this bias using Deeks' funnel plots [14]. All the analyses were performed using the following statistical software programs: Meta-Disc 1.4 for Windows (XI Cochrane Colloquium) and STATA, version 12.0 (Stata Corporation), a two-sided *P*<0.05 was considered statistically significant.

## RESULTS

### Clinical characteristics of included studies

After a systematic literature search and selection, a total of nine studies with 1214 subjects were included in this meta-analysis [15–23]. The article selection process used in the present study is summarized in Figure 1. There were 357 patients with SNPT and 857 controls. Seven studies performed in Asia [15–17,19–22] and two studies performed in Europe [18,23]. All studies provided the detailed diagnostic criteria for SNPT, including bacteriology, histopathology or clinical diagnosis, which was widely accepted in studies with tuberculosis diagnosis. Target sequences for NAAT were given in all studies except for one study [15]. There were six studies with QUADAS scores greater than 9, suggesting the relative high quality of included studies. The detailed information of included studies and QUADAS scores of each study were listed in Table 1.

**Figure 1:**
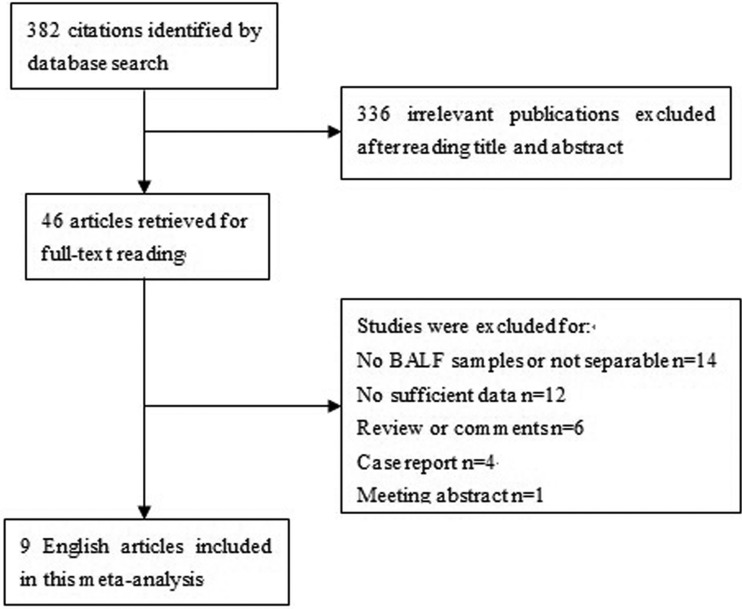
Flow chart of selection process for eligible articles

**Table 1:** Clinical summary of included studies Abbreviations: Bac, bacteriology; Clin, clinical diagnosis; FN, false negative; FP, false positive; His, histopathology; TBC, tuberculosis complex; TN, true negative; TP, true positive.

Author (Ref)	Year	Country	Subjects	Reference standard	NAAT type	Target sequence	TP	FP	FN	TN	Design	QUADAS
Boonsarngsuk et al. [15]	2012	Thailand	424	Bac or His or Clin	Multiplex TBC PCR	Not reported	34	3	87	300	prospective	12
Chou et al. [16]	2012	China	105	Bac or Clin	Amplified *Mycobacterium tuberculosis* direct test	*Amplicor MTB*	20	2	5	78	retrospective	11
Lee et al. [17]	2011	Korea	99	Bac or His	Real-time PCR	IS6110	21	5	6	67	retrospective	11
Jafari et al. [18]	2009	Germany/Netherlands/Italy/Spain	248	Bac or Clin	In house PCR	IS6110	16	6	40	186	prospective	10
Chen et al. [19]	2002	China	40	Bac or Clin	Regular PCR	IS6110	5	1	9	25	retrospective	9
Hidaka et al. [20])	2000	Japan	96	Bac	Regular PCR	65 kDa antigen	17	6	5	68	retrospective	9
Liam et al. [21]	1998	Malaysia	103	Bac or His or Clin	Regular PCR	IS986	55	2	13	33	prospective	7
Tan et al. [22]	1997	Singapore	47	Bac or Clin	Regular PCR	IS6110	15	4	0	28	prospective	6
Brugiere et al. [23]	1997	France	52	Clin	Regular PCR	IS6110	9	0	0	43	retrospective	6

### Diagnostic accuracy

Heterogeneity examination suggested that the *χ*^2^-values of sensitivity, specificity, PLR, NLR and DOR were 120.32, 63.2, 8.89, 91.65 and 13.57, with *P*-values of sensitivity, specificity and DOR less than 0.05, suggesting a significant heterogeneity among included studies. Thus, a random-effect model was chosen to synthesize data. The pooled sensitivity of BALF NAATs was 0.54 [95% CI (confidence interval): 0.48–0.49; Figure 2) and the pooled specificity was 0.97 (95% CI: 0.95–0.98; Figure 3). The PLR and NLR were 12.13 (95% CI: 8.23–17.88) and 0.36 (95% CI: 0.23–0.56) respectively. The overall DOR was 44.71 (95% CI: 22.30–89.63; Figure 4).

**Figure 2:**
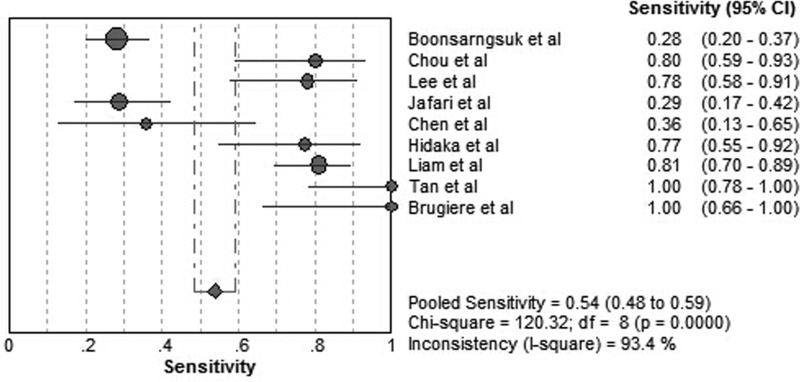
Forest plots of the sensitivity of BALF–NAAT for the diagnosis of SNPT The circles and the horizontal lines represent the point estimate and 95% CI for each included study and the diamond represents the pooled estimate.

**Figure 3:**
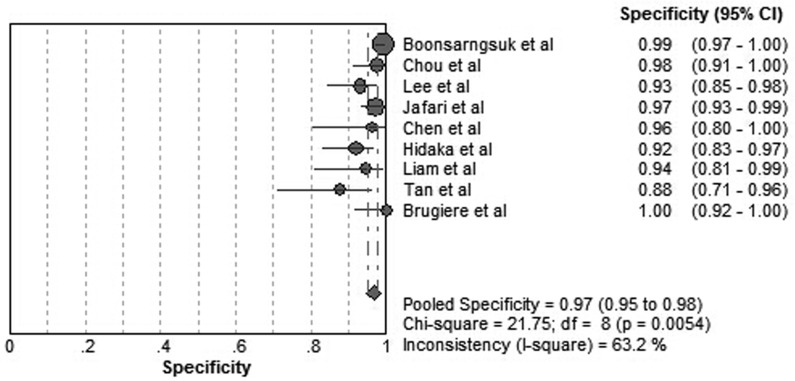
Forest plots of the specificity of BALF–NAAT for the diagnosis of SNPT The circles and the horizontal lines represent the point estimate and 95% CI for each included study and the diamond represents the pooled estimate.

**Figure 4:**
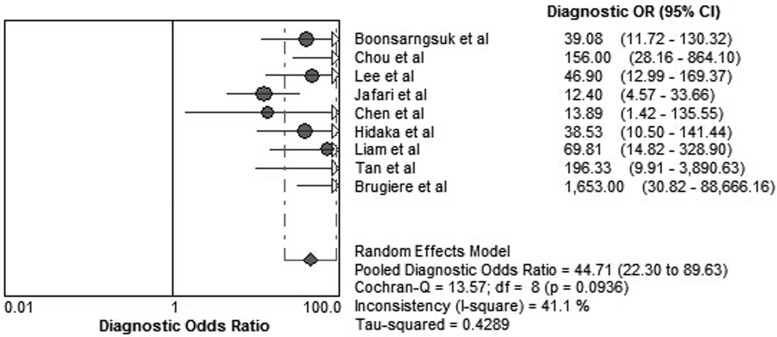
Forest plots of the DOR of BALF–NAAT for the diagnosis of SNPT The circles and the horizontal lines represent the point estimate and 95% CI for each included study and the diamond represents the pooled estimate.

The SROC curve shows an overall summary of tests, which illustrates the relationship between sensitivity and specificity. As shown in Figure 5, the AUC was 0.96 and the Q* was 0.91, indicating a high diagnostic accuracy. Figure 6 shows the Fagan's nomogram for likelihood ratios and the results indicated that the BALF NAATs for detection SNPT increased the post-probability to 82% when the results were positive and reduced the post-probability to 7% when the results were negative

**Figure 5:**
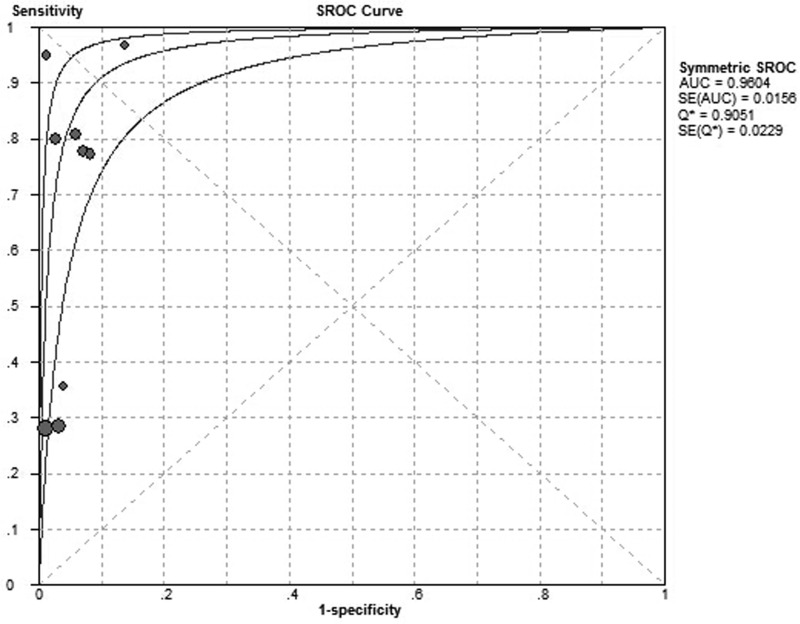
SROC curves for BALF–NAAT in the diagnosis of SNPT

**Figure 6:**
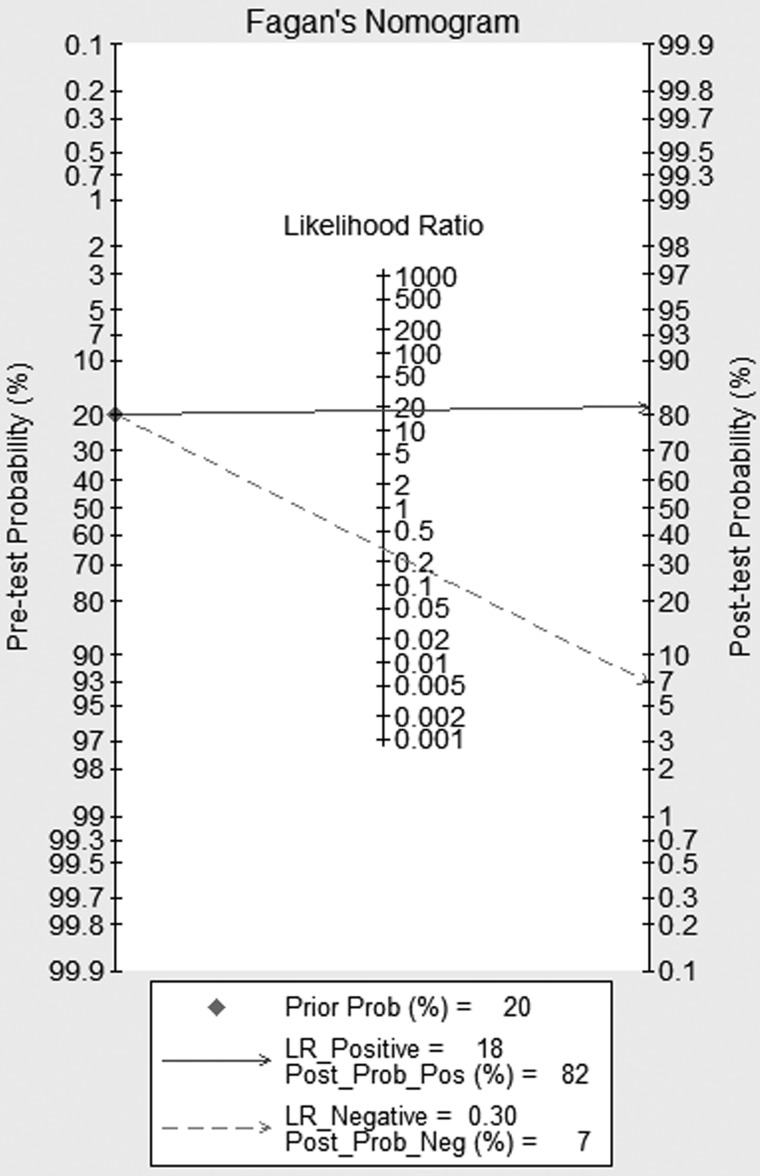
Fagan's nomogram for likelihood ratios and pre- and post-test probabilities of BALF–NAAT for the diagnosis of SNPT

### Meta-regression and publication bias

Since significant heterogeneity was identified among included studies, a meta-regression analysis was performed to explore the possible covariates for the heterogeneity. We selected four co-variates in the present meta-regression: ethnicity (Asian compared with European), sample size (≥100 compared with <100), study design (prospective compared with retrospective) and QUADAS scores (≥9 compared with <9). The outcomes of the regression were shown in Table 2. In the present study, none of the above covariates were found to be the significant source of heterogeneity (all *P* > 0.05). According to the Deeks' funnel plot asymmetry test, the statistically non-significant value (*P* = 0.16) for the slope coefficient suggests symmetry in the data and a low likelihood of publication bias (Figure 7).

**Table 2:** Meta-regression of potential heterogeneity within the included studies

Co-variates	Number of studies	Coefficient	S.E.M.	RDOR (95% CI)	*P*-value
Ethnicity					
Asian	7	0.544	0.6988	1.72 (0.19–15.93)	0.4929
European	2				
Sample size					
≥100	4	0.836	0.9275	2.31 (0.12–44.15)	0.4339
<100	5				
Design					
Prospective	4	−1.467	1.1174	0.23 (0.01–8.08)	0.2807
Retrospective	5				
QUADAS					
≥9	6	−1.769	1.3403	0.17 (0.00–12.14)	0.2786
<9	3				

**Figure 7:**
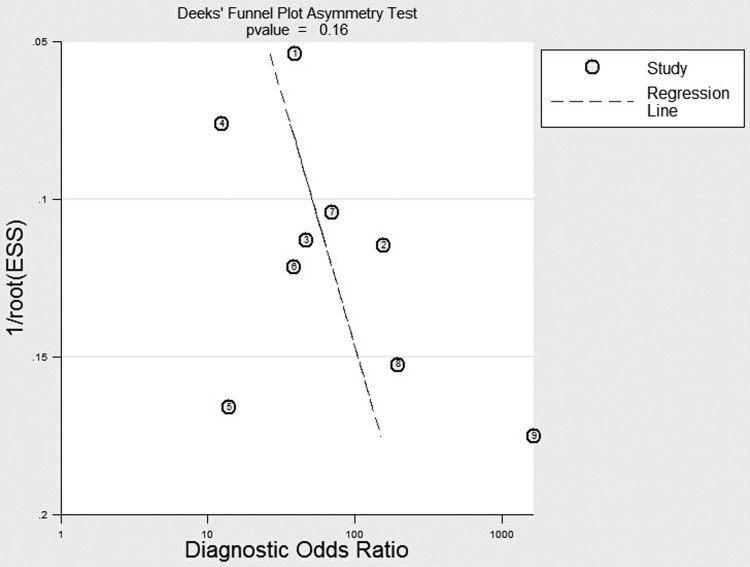
Linear regression test of funnel plot asymmetry The statistically non-significant value (*P* = 0.16) for the slope coefficient suggests symmetry in the data and a low likelihood of publication bias.

## DISCUSSION

Lack of accurate and rapid diagnostic methods for pulmonary tuberculosis has been a major problem for global tuberculosis control especially for SNPT [24]. NAATs were introduced as hopeful novel tests for pulmonary tuberculosis and a lot of commercial assays were introduced into the market [25]. In recent years, many diagnostic tests have focused on the value of NAATs on BALF for the diagnosis of SNPT, but the results remain controversial, as meta-analysis is an important tool for accurately and reliably summarizing evidence, we performed this study to comprehensively assess the overall diagnostic accuracy of NAATs on BALF for SNPT.

Our study found that the sensitivity and specificity of NAATs on BALF for the diagnosis of SNPT were 0.54 and 0.97 respectively, which demonstrated that the sensitivity of NAATs on BALF was low and might limit the clinical utility as a screening tool for SNPT. However, the specificity of 0.97 is relatively high to confirm diagnosis. The SROC curve presents as a summary of the diagnostic performance, which shows the trade-off between sensitivity and specificity [26]. Our SROC analysis showed that the maximum joint of sensitivity and specificity was 0.91 and an AUC of 0.96, suggesting a high overall accuracy.

DOR, defined as the ratio of the odds of a true positive to the odds of a false positive, is a single indicator of test performance that combines the data of sensitivity and specificity into a single number [27]. The value of a DOR ranges from zero to infinity and a higher value means a higher diagnostic accuracy. In our meta-analysis, the pooled DOR was 44.71, suggesting that NAATs on BALF seemed to be helpful in the diagnosis of SNPT. However, the SROC curve and the DOR are not convenient to interpret and utilize in clinical practice, whereas the likelihood ratios (PLR and NLR) are considered more clinically meaningful. A PLR value of 10.307 suggests that patients with SNPT have about 10-fold higher chance having a positive NAATs results than those without. Meanwhile, the pooled NLR was 0.348, which means that the probability of having SNPT in NAATs-negative patients is about 35%. Therefore, BALF NAAT results should be interpreted in parallel with clinical findings and the results of conventional tests.

NAATs have been used for pulmonary tuberculosis diagnosis for a long time [28], but according to our study, two problems should be noticed when applied in SNPT diagnosis. First, the pooled sensitivity of NAATs was only 0.54, with extremely varying values in different studies. Thus, NAAT may not be a satisfactory screening tool for SNPT and further studies should pay attention to the clinical background and several other factors under which NAATs are conducted to better explain such variability. Second, the procedure of NAATs is invasive; it may not be widely used in facilities at all levels and well tolerated. We recommend that the traditional examinations, such as sputum smear/culture, skin test, chest imaging and other tests should also be considered when establishing the diagnosis of SNPT, as combination of NAATs and other examinations would increase the diagnostic accuracy [16,19] and NAATs in BALF should not replace conventional diagnostic approaches.

There were several limitations in our study that should be addressed. First, only published studies were included in our meta-analysis, whereas unpublished data and conference abstracts were not sought, which probably leads to publication bias and affects diagnostic accuracy estimates. What should also be pointed out is that although Deeks' funnel plot asymmetry test suggested a low likelihood of publication bias, the power of this test might be low due to the small number of studies included. Hence, publication bias cannot really be excluded. Second, potential heterogeneity was recognized among included studies. However, meta-regression didn't identify covariates affecting the diagnostic accuracy. Therefore, further studies should pay attention to these aspects. Third, we only included English articles, which probably cause language bias.

In summary, based on current available evidences, NAATs in BALF play a role in confirming diagnosis of SNPT. Nevertheless, more studies should be carried out to validate our findings. It is also desirable to conduct studies using more rigid methods to appreciate better diagnostic performance of the NAATs.

## Abbreviations

AUC: area under the curve
BALF: bronchoalveolar lavage fluid
CI: confidence interval
DOR: diagnostic odds ratio
NAAT: nucleic acid amplification test
NLR: negative likelihood ratio
PLR: positive likelihood ratio
QUADAS: Quality Assessment for Studies of Diagnostic Accuracy
SNPT: smear-negative pulmonary tuberculosis
SROC: summary receiver operating characteristic

## References

[1] World Health Organization (2013). Global Tuberculosis Report.

[2] Shah N.S., Cavanaugh J.S., Pratt R., Cain K.P., Wells C., Laserson K., Nelson L. (2012). Epidemiology of smear-negative pulmonary tuberculosis in the United States, 1993-2008. Int. J. Tuberc. Lung. Dis..

[3] Steingart K.R., Ng V., Henry M, Ramsay A., Cunningham J., Urbanczik R., Perkins M.D., Aziz M.A., Pai M. (2006). Sputum processing methods to improve the sensitivity of smear microscopy for tuberculosis: a systematic review. Lancet. Infect. Dis..

[4] Swai H.F., Mugusi F.M., Mbwambo J.K. (2011). Sputum smear negative pulmonary tuberculosis: sensitivity and specificity of diagnostic algorithm. BMC Res. Notes.

[5] Van Staden M., van der Ryst E., Attwood E. M., Hendricks M.L., Joubert G., Weich D.J. (1998). Detection of *Mycobacterium tuberculosis* in serum samples using the polymerase chain reaction. J. Infect. Dis.

[6] Tan J., Lee B.W., Lim T.K., Chin N.K., Tan C.B., Xia J.R., Yap H.K., Kumarasinghe G. (1995). Detection of Mycobacterium tuberculosis in sputum, pleural, and bronchoalveolar lavage fluid using DNA amplification of the MPB 64 protein coding gene and IS6110 insertion element. Southeast Asian J. Trop. Med. Public. Health.

[7] Lin J.J., Harn H.J., Hsu Y.D., Tsao W.L., Lee H.S., Lee W.H. (1995). Rapid diagnosis of tuberculous meningitis by polymerase chain reaction assay of cerebrospinal fluid. J. Neurol..

[8] Yeh T.S., Chen N.H., Jan Y.Y., Hwang T.L., Jeng L.B., Chen M.F. (1999). Obstructive jaundice caused by biliary tuberculosis: spectrum of the diagnosis and management. Gastroint. Endo..

[9] Greco S., Rulli M., Girardi E., Piersimoni C., Saltini C. (2009). Diagnostic accuracy of in-house PCR for pulmonary tuberculosis in smear-positive patients: meta-analysis and metaregression. J. Clin. Microbiol..

[10] Ramírez P., Valencia M., Torres A. (2007). Bronchoalveolar lavage to diagnose respiratory infections. Semin. Respir. Crit. Care. Med..

[11] Virgili G., Conti A.A., Murro V., Gensini G.F., Gusinu R. (2009). Systematic reviews of diagnostic test accuracy and the Cochrane collaboration. Intern. Emerg. Med..

[12] Whiting P.F., Weswood M.E., Rutjes A.W., Reitsma J.B., Bossuyt P.N., Kleijnen J. (2006). Evaluation of QUADAS, a tool for the quality assessment of diagnostic accuracy studies. BMC Med. Res. Methodol..

[13] Shen Y., Zhu H., Wan C., Chen L., Wang , T., Yang T., Wen F. (2014). Can cholesterol be used to distinguish pleural exudates from transudates? evidence from a bivariate meta-analysis. BMC Pulm. Med.

[14] Deeks J.J., Macaskill P., Irwig L. (2005). The performance of tests of publication bias and other sample size effects in systematic reviews of diagnostic test accuracy was assessed. J. Clin. Epidemiol..

[15] Boonsarngsuk V., Suwannaphong S., Laohavich C. (2012). Combination of adenosine deaminase activity and polymerase chain reaction in bronchoalveolar lavage fluid in the diagnosis of smear-negative active pulmonary tuberculosis. Int. J. Infect. Dis..

[16] Chou P.C., Wang C.H., Huang C.D., Lo Y.L., Lee K.Y., Yu C.T., Lin S.M., Lin H.C., Kuo H.P. (2012). Nucleic acid amplification test and bronchoscopy improve the diagnostic accuracy of smear-negative tuberculosis. Int. J. Tuberc. Lung. Dis..

[17] Lee J.E., Lee B.J., Roh E.Y., Kim D.K., Chung H.S., Lee C.H. (2011). The diagnostic accuracy of tuberculosis real-time polymerase chain reaction analysis of computed tomography-guided bronchial wash samples. Diagn. Microbiol. Infect. Dis..

[18] Jafari C., Thijsen S., Sotgiu G., Goletti D., Domínguez Benítez J.A., Losi M., Eberhardt R., Kirsten D., Kalsdorf B. (2009). Bronchoalveolar lavage enzyme-linked immunospot for a rapid diagnosis of tuberculosis: a Tuberculosis Network European Trials group study. Am. J. Respir. Crit. Care Med..

[19] Chen N.H., Liu Y.C., Tsao T.C., Wu T.L., Hsieh M.J., Chuang M.L., Huang C.C., Kuo A.J., Chen M.C., Yang C.T. (2002). Combined bronchoalveolar lavage and polymerase chain reaction in the diagnosis of pulmonary tuberculosis in smear-negative patients. Int. J. Tuberc. Lung. Dis..

[20] Hidaka E., Honda T., Ueno I., Yamasaki Y., Kubo K., Katsuyama T. (2000). Sensitive identification of mycobacterial species using PCR-RFLP on bronchial washings. Am. J. Respir. Crit. Care. Med..

[21] Liam C.K., Chen Y.C., Yap S.F., Srinivas P., Poi P.J. (1998). Detection of *Mycobacterium tuberculosis* in bronchoalveolar lavage from patients with sputum smear-negative pulmonary tuberculosis using a polymerase chain reaction assay. Respirology.

[22] Tan M.F., Ng W.C., Chan S.H., Tan W.C. (1997). Comparative usefulness of PCR in the detection of Mycobacterium tuberculosis in different clinical specimens. J. Med. Microbiol..

[23] Brugière O., Vokurka M., Lecossier D., Mangiapan G., Amrane A., Milleron B., Mayaud C., Cadranel J., Hance A.J. (1997). Diagnosis of smear-negative pulmonary tuberculosis using sequence capture polymerase chain reaction. Am. J. Respir. Crit. Care. Med..

[24] Siddiqi K., Lambert M.L., Walley J. (2003). Clinical diagnosis of smear-negative pulmonary tuberculosis in low-income countries: the current evidence. Lancet Infect. Dis..

[25] Ling D.I., Flores L.L., Riley L.W., Pai M. (2008). Commercial nucleic-acid amplification tests for diagnosis of pulmonary tuberculosis in respiratory specimens: meta-analysis and meta-regression. PLoS One.

[26] Jones C.M., Athanasiou T. (2005). Summary receiver operating characteristic curve analysis techniques in the evaluation of diagnostic tests. Ann. Thorac. Surg..

[27] Glas A.S., Lijmer J.G., Prins M.H., Bonsel G.J., Bossuyt P.M. (2003). The diagnostic odds ratio: a single indicator of test performance. J. Clin. Epidemiol..

[28] Grecom S., Girardi E., Navarra A., Saltini C. (2006). Current evidence on diagnostic accuracy of commercially based nucleic acid amplification tests for the diagnosis of pulmonary tuberculosis. Thorax.

